# Synergic effect of adsorbed gas and charging on surface flashover

**DOI:** 10.1038/s41598-019-41961-0

**Published:** 2019-04-02

**Authors:** Shengtao Li, Yin Huang, Daomin Min, Guanghao Qu, Huan Niu, Zhen Li, Weiwang Wang, Jianying Li, Wenfeng Liu

**Affiliations:** State Key Laboratory of Electrical Insulation and Power Equipment, Xi’an Jiaotong University, Xi’an, 710049 China

## Abstract

Flashover is a crucial issue in both high-voltage engineering and surface physics. It not only challenges the existing theories about its dynamic evolution, but also inhibits the clean energy revolution by limiting the accessible voltage rating of power equipment. It is of significance to elucidate the microscopic process along the interface to improve the flashover performance. In the present study, the synergic effect of adsorbed gas and surface charging is investigated, which reveals a long ignored factor for determining the flashover voltage. Depending on the relative amount of adsorbed gas, the flashover voltage varies, which exhibit different behavior from the bulk breakdown of the same gas. The amount of N_2_ gas adsorbed on epoxy resin (EP) surface is much larger than that on Al_2_O_3_ ceramic surface, corresponding to the observed higher flashover voltage on EP. It is proposed that the adsorbed gas molecules not only modify the local surface charging state via their interaction with the trapped charges, but also capture free electrons due to the distortion of their electronic distribution. Both effects suppress the free path length of electrons in the gas-solid interface. This work explores another possibility to improve the surface flashover performance.

## Introduction

Flashover in ultrahigh voltage power equipment is one of such challenges, which significantly affects the stability and reliability of the system^1–5^. The flashover is a continuous electrical breakdown process from cathode to anode, which propagates along the interface between gas and insulator^1^. The flashover voltage is defined as the voltage when the first flashover is observed, which is much lower than the bulk breakdown voltage of either pure gas or insulator with the same inter-electrode distance. In other words, the flashover undermines the insulating level of the system, which is indeed encountered in the ultrahigh voltage system^6,7^. Actually, even in low voltage system, the flashover is not rare especially in cases with severely distorted electric field distribution. With the development of high power and small scale electronics, flashover becomes increasingly important^8–11^. In addition, flashover not only induces temporal electrical breakdown, but also generates high frequency electromagnetic noises, arcs and high temperature, which damage the equipment and devices permanently.

The surface flashover involves the production of initial electrons and the accumulation of surface charges, as well as the development of electron avalanche and formation of discharge channels on solid surface^1,5,12^. Although flashover is a complicated process that is altered by applied voltage waveform, electrode structure, solid properties, gas species, temperature and others, the surface charging is proposed to be the most critical one^13–17^, which is the key point in various theoretical models including critical field theory^18^, the diffusion-limited charge accumulation model^19^ and the comprehensive analytical model^20^. On one hand, the surface charging varies the electric field distribution across the interface, affecting the charge transport. On the other hand, its properties depend on both the solid insulator and gas. Nevertheless, there is still no consensus on the following experimental observations, i.e., (1) gas pressure dependence of the flashover voltage and its relation with the bulk gas breakdown voltage; (2) the spatial location of the flashover path, (3) the critical role of the gas-insulator interface. Without clarifying those problems, it is not possible to correctly understand and effectively improve the flashover behaviour.

In the present work, it is found that the synergic effect of adsorbed gas and surface charging plays an important role in determining the flashover voltage, which is a long ignored factor. The adsorption is confirmed experimentally in two different insulators, i.e., Al_2_O_3_ and epoxy resin (EP), which reveals a higher gas adsorption on the EP surface. The adsorbed gas can not only modify the local surface charging state via their interaction with the trapped charges, but also capture free electrons due to the distortion of their electronic distribution. Both effects reduce the free path length of electrons in the gas-solid interface, resulting in an enhancement of flashover voltage. The higher flashover voltage measured in EP indicates the validity of the proposed model. The synergic effect is observed in several gas environments and further confirmed by DFT calculations. The present work offers a new insight into the mechanism of flashover, which is of help for further understanding and manipulating flashover.

## Results

### Displacement of the lowest point in gas breakdown and surface flashover curve

**Figure 1:**
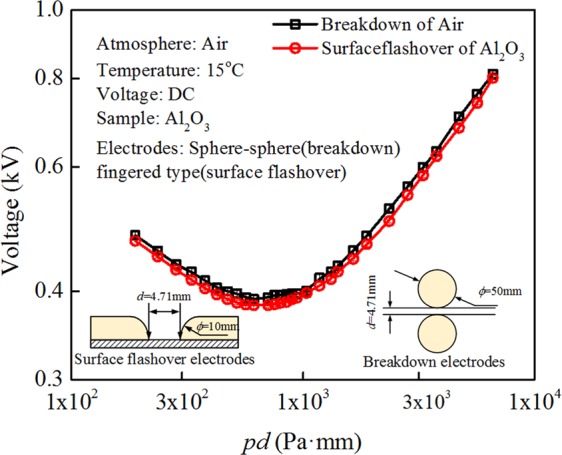
The shift of the lowest point in gas breakdown and surface flashover voltages.

Figure 1 shows the experimental results of electrical breakdown voltage of an air gap and the surface flashover voltage across an alumina dielectric with respect to *pd* value, here, *p* is the pressure and *d* is the inter-electrode distance. Both experiments are conducted in the same chamber and environments. The electrode system for each experiment and the measurement conditions are exhibited as insets in Fig. 1. During both experiments, the inter-electrode distance is kept the same of 4.71 mm, while the gas pressure is varied from 40 to 1400 Pa. The typical Paschen curves are observed, i.e., for high and low *pd*, both the breakdown and flashover voltages are large, while it exhibits a minimum at 759 Pa·mm and 622 Pa·mm for gas breakdown and surface flashover, respectively. In Fig. 1, it is shown that the breakdown voltage of an air gap is higher than the flashover voltage within the measurement range. Nevertheless, the minimum position of surface flashover voltage is shifted to lower value compared with the breakdown voltage. Considering the flashover is essentially a gas breakdown process above the solid-gas interface, the displacement of the lowest position in *pd* implies a local environment, especially the gas molecule status difference between two cases, which will be elaborated below.

### Adsorption of N_2_ on epoxy resin and Al_2_O_3_ particles

**Figure 2:**
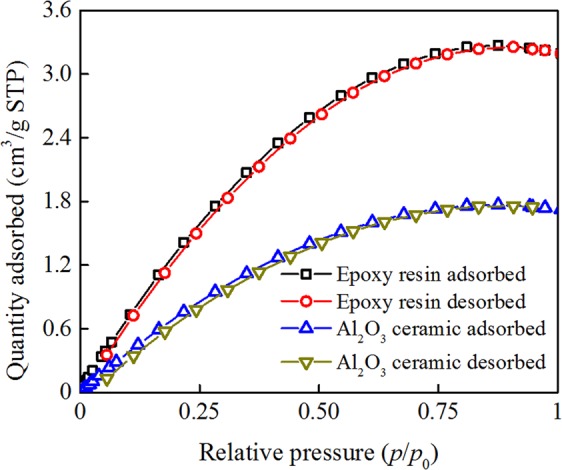
Isothermal adsorption and desorption curves for neat epoxy resin and Al_2_O_3_ ceramic at room temperature (25 °C). STP stands for the standard temperature and pressure, *p*_0_ is the saturated vapor pressure with the value of 760 mmHg, and *p* is the equilibrium vapor pressure.

Figure 2 shows the isothermal adsorption and desorption curves of neat epoxy resin and Al_2_O_3_ ceramic particles in micrometer scale at room temperature (25 °C). The curves represent a reversible adsorption process that occurs on a non-porous solid surface or a macro porous solid surface^21^. In Fig. 2, the curves are convex upward, reflecting that the adsorbate (N_2_) interacts with the adsorbent (epoxy resin and Al_2_O_3_ ceramic particles). It can be seen from Fig. 2 that the adsorption and desorption quantity of N_2_ on neat epoxy resin and Al_2_O_3_ ceramic firstly increases with an increase in *p*/*p*_0_ and then approaches saturation. In the full measurement range, the adsorption quantity of N_2_ on neat epoxy resin is significantly larger than that on Al_2_O_3_ ceramic. It indicates that the N_2_ adsorption capability on neat epoxy resin is greater than that on Al_2_O_3_ ceramic at room temperature.

### Gas breakdown and surface flashover voltage

**Figure 3:**
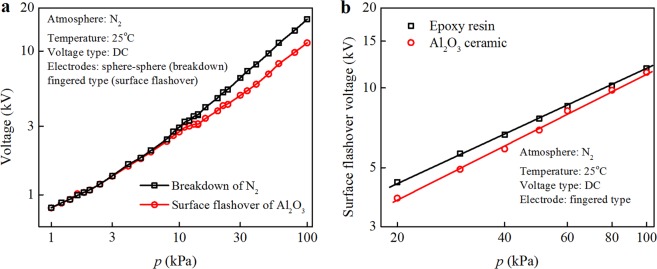
Comparison between gas breakdown and surface flashover voltage for Al_2_O_3_ ceramic and neat epoxy resin at room temperature. (**a**) Al_2_O_3_ ceramic and (**b**) the comparison of surface flashover voltage for Al_2_O_3_ ceramic and neat epoxy resin.

The breakdown voltage of N_2_ and the surface flashover voltage of Al_2_O_3_ ceramic and neat epoxy resin varies with pressures at room temperature, as shown in Fig. 3. A conventional two parameters Weibull distribution is used to fit the experimental results. In Fig. 3a, both the gas breakdown voltage and the surface flashover voltage increase with gas pressure, and the former is greater than the latter one. Above 20 kPa, the difference value between the gas breakdown and the surface flashover voltage increases. A comparison between the surface flashover voltage of neat epoxy resin and that of Al_2_O_3_ ceramic at various gas pressures is shown in Fig. 3b. It can be seen that the surface flashover voltage of neat epoxy resin is greater than that of Al_2_O_3_ ceramic in the range of 20 kPa to 100 kPa. As mentioned above, the adsorption quantity of N_2_ on epoxy resin is larger than that on Al_2_O_3_ ceramic at room temperature, implying a direct influence of gas absorption quantity on the flashover voltage, i.e., the more the gas adsorption quantity, the larger the surface flashover voltage.

### Relation between the ratio of surface flashover voltage to breakdown voltage and gas pressure

**Figure 4:**
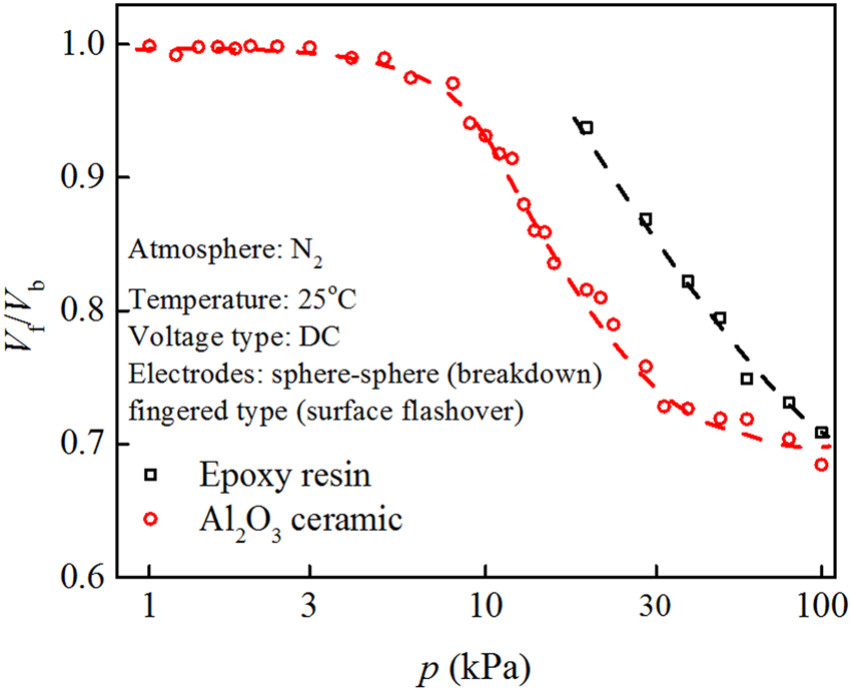
The relationship between *V*_f_/*V*_b_ and *p* for neat epoxy resin and Al_2_O_3_ ceramic.

Figure 4 shows the relationship between the ratio of dc surface flashover voltage *V*_f_ and gas breakdown voltage *V*_b_ in N_2_ and gas pressure for neat epoxy resin and Al_2_O_3_ ceramic at room temperature. For the Al_2_O_3_ ceramic sample, *V*_f_/*V*_b_ is close to 1 at *p* < 10 kPa and decreases as gas pressure increases. In the range of 10 kPa to 35 kPa, *V*_f_/*V*_b_ decreases rapidly. At *p* > 35 kPa, *V*_f_/*V*_b_ is still slowly decreasing. For neat epoxy resin, *V*_f_/*V*_b_ tends to decrease with increasing gas pressure *p*, and has not dropped to the lowest point when *V*_f_/*V*_b_ is about 0.7. It shows that the gas pressure *p* has an effect on gas breakdown and surface flashover, but the degrees of influence are different.

It can also be seen from Fig. 4 that the value of *V*_f_/*V*_b_ of neat epoxy resin is greater than 0.9 at 20 kPa, while the value of *V*_f_/*V*_b_ of Al_2_O_3_ ceramic drops to about 0.8 above 20 kPa. The gas adsorption capabilities of two materials are different, affecting the ratio of flashover voltage to gas breakdown voltage. At the same pressure, the N_2_ adsorption quantity on Al_2_O_3_ ceramic is smaller than that on neat epoxy resin, and the value of *V*_f_/*V*_b_ of Al_2_O_3_ is smaller than that of neat epoxy resin, which is shown in Fig. 4.

**Figure 5:**
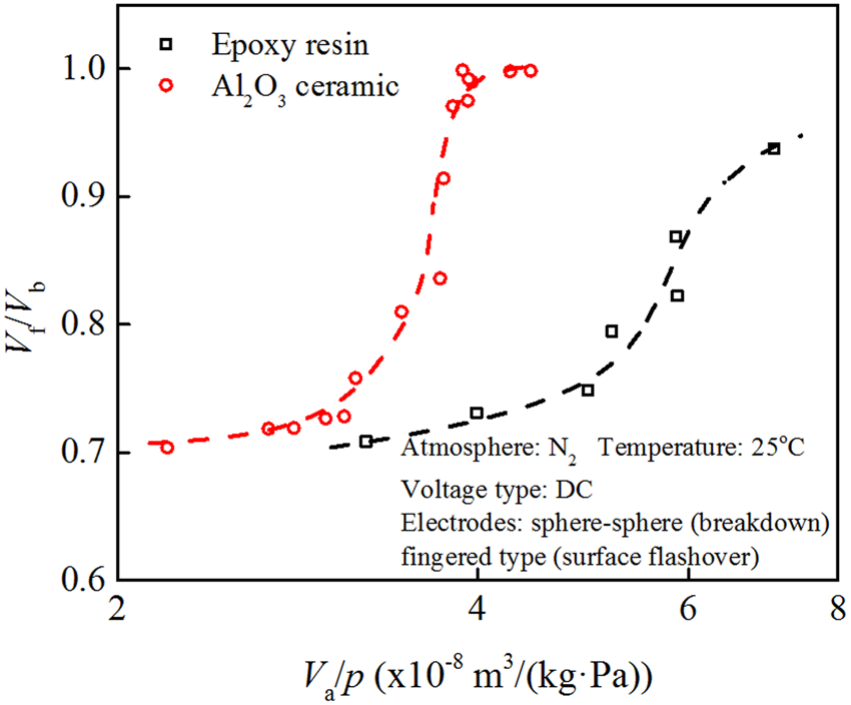
The relationship between *V*_f_/*V*_b_ and the relative adsorption quantity *V*_a_/*p* for neat epoxy resin and Al_2_O_3_ ceramic at room temperature.

The relationship between the value of *V*_f_/*V*_b_ in Fig. 4 and the ratio of gas adsorption quantity *V*_a_ in Fig. 2 to pressure *p* (labeled as *V*_a_/*p*) for neat epoxy resin and Al_2_O_3_ ceramic is shown in Fig. 5.

For neat epoxy resin, *V*_f_/*V*_b_ increases approximate linearly with the increase in the relative adsorption quantity *V*_a_/*p*. For Al_2_O_3_ ceramic, *V*_f_/*V*_b_ increases slowly at first and then sharply with the increase in relative adsorption quantity *V*_a_/*p* and ultimately reaches a stable value around 1. At 35 kPa or more, *V*_f_/*V*_b_ is about 0.7. When the gas pressure is lower than 5 kPa, *V*_f_/*V*_b_ is about 0.99.

Since *V*_a_ represents the adsorption volume per unit mass and *p* is positively correlated with the average gas concentration at a certain temperature, *V*_a_/*p* can approximately reflect the ratio of the concentration (*N*_surface_) of gas adsorption layer to the gas concentration (*N*_bulk_) in the space above the solid surface. Figure 5 can be regarded as the relationship between *V*_f_/*V*_b_ and *N*_surface_/*N*_bulk_, which reflects the influence of the gas adsorption on surface flashover voltage. As the relative adsorption quantity (*N*_surface_/*N*_bulk_) increases, the value of *V*_f_/*V*_b_ increases, indicating that gas adsorption affects surface flashover voltage. In the case that gas pressure is not high, the relative adsorption quantity is large, the gas adsorption quantity is high relative to the gas concentration in the space above the solid surface, and the flashover voltage is approaching the gas breakdown voltage. With a decrease in gas pressure, surface flashover voltage verges on gas breakdown voltage, and the relative adsorption quantity is close to 1. The impact ionization process in the gas adsorption layer is weaker at low pressure than that at high pressure.

Comparing the two curves in Fig. 5, the relationship between the relative voltage ratio *V*_f_/*V*_b_ and relative adsorption quantity *V*_a_/*p* for different samples differs from each other. Although the relative voltage ratios of neat epoxy resin and Al_2_O_3_ ceramic samples increase with an increase in relative adsorption quantity, both the increasing trend and the pressure range with a noticeable change in relative voltage ratio for the two samples are different. The pressure range with a dramatic change in relative voltage ratio for Al_2_O_3_ ceramic sample is narrower than that for neat epoxy resin. It shows that in a wide pressure range the gas adsorption process of epoxy resin will significantly affect the flashover voltage, while the effect of gas adsorption for Al_2_O_3_ ceramic on its surface flashover voltage is obvious in the pressure range of 5 kPa–35 kPa. Therefore, controlling the gas adsorption process on solid material can change the surface flashover voltage.

### Calculation of adsorption layer concentration and surface flashover voltage

**Figure 6:**
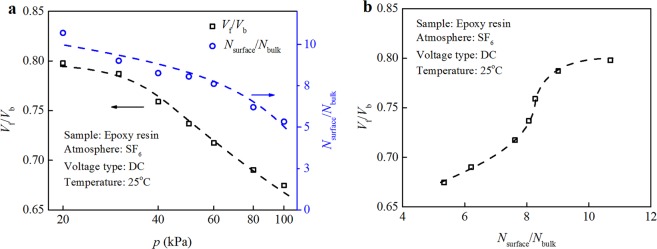
Relationship between *V*_f_/*V*_b_ and *N*_surface_/*N*_bulk_ for neat epoxy resin. (**a**) Relationship between *V*_f_/*V*_b_ and *N*_surface_/*N*_bulk_ and gas pressure and (**b**) Relationship between *V*_f_/*V*_b_ and *N*_surface_/*N*_bulk_.

Figure 6 shows the relationship between the ratio of surface flashover voltage and gas breakdown voltage *V*_f_/*V*_b_ and the ratio of concentration of gas adsorption layer and the gas equilibrium concentration *N*_surface_/*N*_bulk_ for epoxy resin in SF_6_ atmosphere. It can be seen *V*_f_/*V*_b_ and *N*_surface_/*N*_bulk_ decrease with an increase in gas pressure. It indicates that the relative adsorption quantity *N*_surface_/*N*_bulk_ and relative voltage ratio *V*_f_/*V*_b_ at high pressure are lower than that at low pressure. As the relative adsorption quantity increases, the relative voltage ratio increases and reaches saturation gradually.

**Figure 7:**
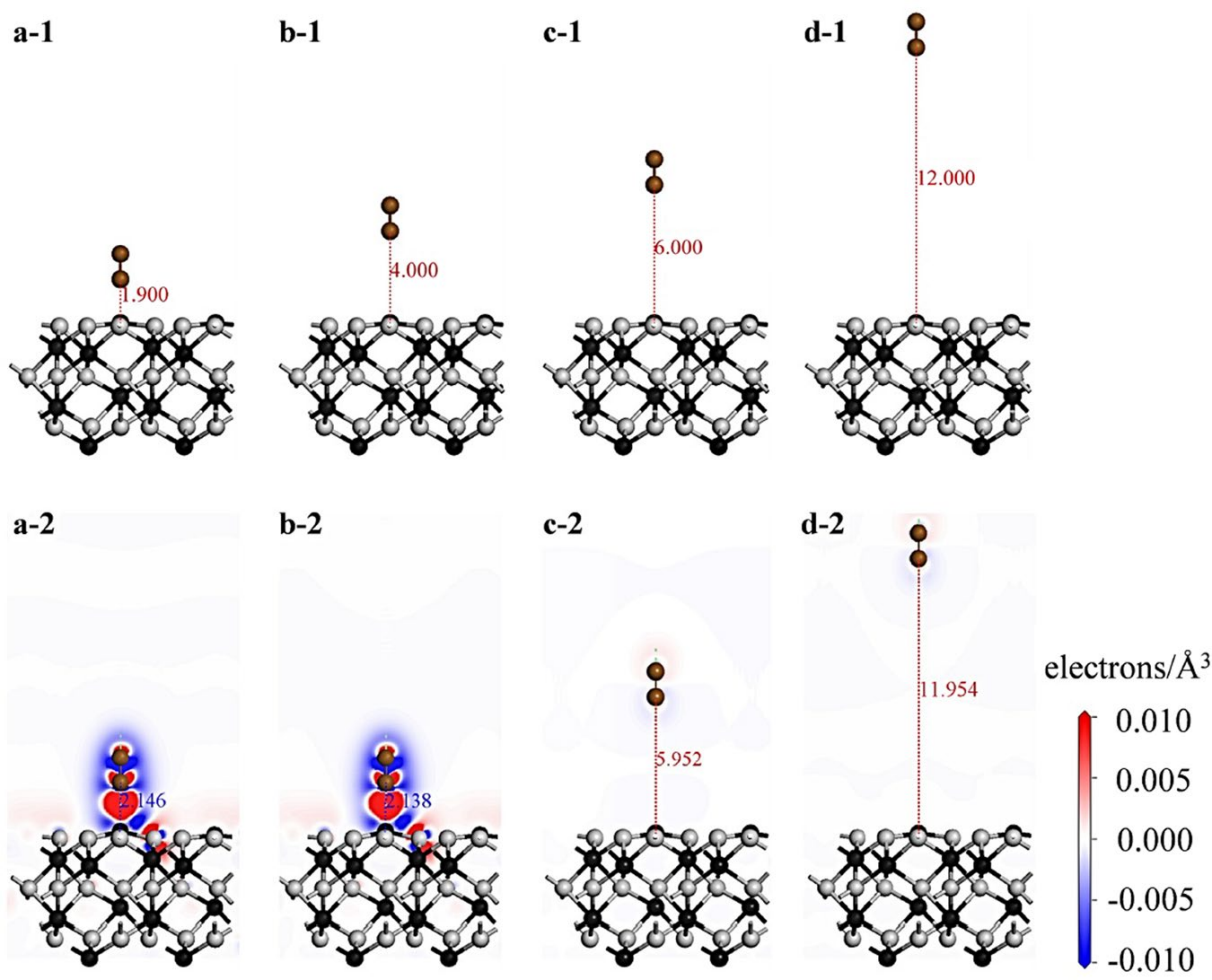
Side view of equilibrium adsorption structures and charge density difference plots of single N_2_ molecule on the α-Al_2_O_3_ (0001) surface with different vertical distance *d*. (**a**) *d* = 2.171Å, (**b**) *d* = 3.977Å, (**c**) *d* = 6.000Å and (**d**) *d* = 12Å. The boundary of boxes was not displayed in these adsorption systems. N, O, and Al atoms are presented by brown, gray, and black spheres. The red areas show where the electron density has been enriched with respect to the fragments, and the blue areas show depleted.

The charge density difference is used to visualize the electron transfer when a gas molecule is adsorbed on a solid surface. The 2-D charge density differences of single N_2_ molecule on the α-Al_2_O_3_ (0001) surface were plotted in Fig. 7. In Figs 7a-2, the red zone was mainly around N atoms, and blue zone around N, and three coordinated surface Al atom near the N_2_/α-Al_2_O_3_ (0001) interface, suggesting that the electrons around the Al are attracted by N atom. The Al-O bond lengths stretch from 1.695Å to 1.705Å, and the distance between Al and N atoms increased to 2.146Å compared to the initial structure. For the initial structure in Figs 7b-1, the distance between Al and N atoms was set 4.000Å, but decreased to 2.138Å after optimizing. The equilibrium structure in Figs 7b-2 is almost like to the adsorption structure in Figs 7a-2. For the adsorption structures with a larger distance (6.000Å and 12.000Å) between Al and N atoms in Figs 7c-1,d-1, the final atomic and electronic structures have little change after geometry optimization.

**Figure 8:**
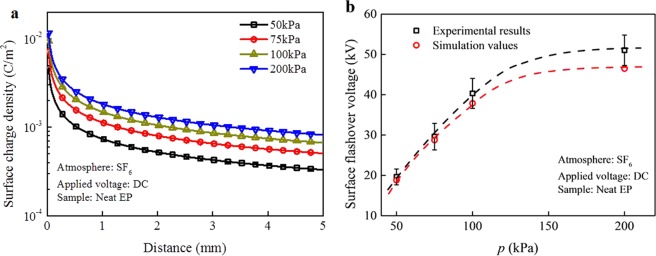
Surface charge density and surface flashover voltage at different gas pressure for neat epoxy resin. (**a**) Calculated surface charge density and (**b**) Surface flashover voltage.

The calculated surface charge density and the surface flashover voltage of neat epoxy resin in SF_6_ atmosphere were calculated under the pressures of 50 kPa, 75 kPa, 100 kPa, and 200 kPa, as shown in Fig. 8. Figure 8a shows the calculated surface charge density of the sample at the moment of the occurrence of surface flashover. As the gas pressure increases, the charge density accumulated near the cathode increases when surface flashover occurs. The calculated surface flashover voltages of neat epoxy resin are shown in Fig. 8b, which exhibit good agreements with the experimental values.

## Discussion

Gas adsorption on solid surface is a ubiquitous and important phenomenon. Not only the properties of solid surface are modified, but the properties of adsorbed molecules themselves vary as well. Recent studies demonstrate a huge decrease in relative dielectric constant of adsorbed water molecule on graphite surface from about 80 to around 2, which is a direct evidence for the molecule-solid surface interaction in a dielectric way^22^. In the present case, the adsorbed molecules have significant effects on the flashover voltage, which is interpreted by a synergic effect of adsorbed molecules and surface charging.

The adsorbed gas molecules form a dense solid-like thin layer, which is confined to several nanometers in the direction normal to the insulator surface (referred to Figs S1 and S3 in Supplementary Information). The properties of the adsorbed gas layer are different from those of the free bulk gas molecules. It is suggested that without considering those adsorbed gas molecules, the breakdown voltage (*V*_b_) should be larger than the flashover voltage (*V*_f_), which is indeed observed in Figs 1 and 3a. Nevertheless, the position of the lowest *pd* point, which is the most favorable condition for breakdown or flashover, shifts between *V*_b_ and *V*_f_ curves, implying some differences in the two processes. Among all the possible reasons, the effect of adsorbed gas molecules is the most likely one. The properties of adsorbed gas molecules are investigated from three aspects, i.e., the amount of adsorbed molecules, the molecular orientation and electronic distribution, which depend on the gas species (N_2_ or SF_6_), gas pressure (1–100 kPa) and solid insulator (EP or Al_2_O_3_).

With more adsorbed gas molecules, the electrons transporting along the interface are scattered more significantly, leading to an enhancement of *V*_f_. The observed higher *V*_f_ of EP than that of Al_2_O_3_ in the whole pressure range verifies well our model. The scattering effect of adsorbed gas molecules on electrons is further enhanced by the specific molecular orientation on the insulator surface as revealed by DFT calculation shown in Fig. 7. From DFT, it is shown that N_2_ molecules in the first several adsorbed layers prefer to exhibit a perpendicular orientation with respect to the insulator surface. In addition, for SF_6_ molecules, the adsorption layer extends to about 4 nm (referred to Fig. S3 in Supplementary Information). Besides the scattering effect, the electronic distribution of the adsorbed molecules is distorted from the symmetric one in the free state to the asymmetric one in the confined state. The distorted electronic distribution shown in Fig. 7 illustrates the separation of positive and negative electronic centers, implying the formation of polarized surface dipoles and strong interaction between gas molecules and solid surface. The surface dipoles capture the mobile electrons in gas near the solid surface, resulting in further inhibition of flashover.

**Figure 9:**
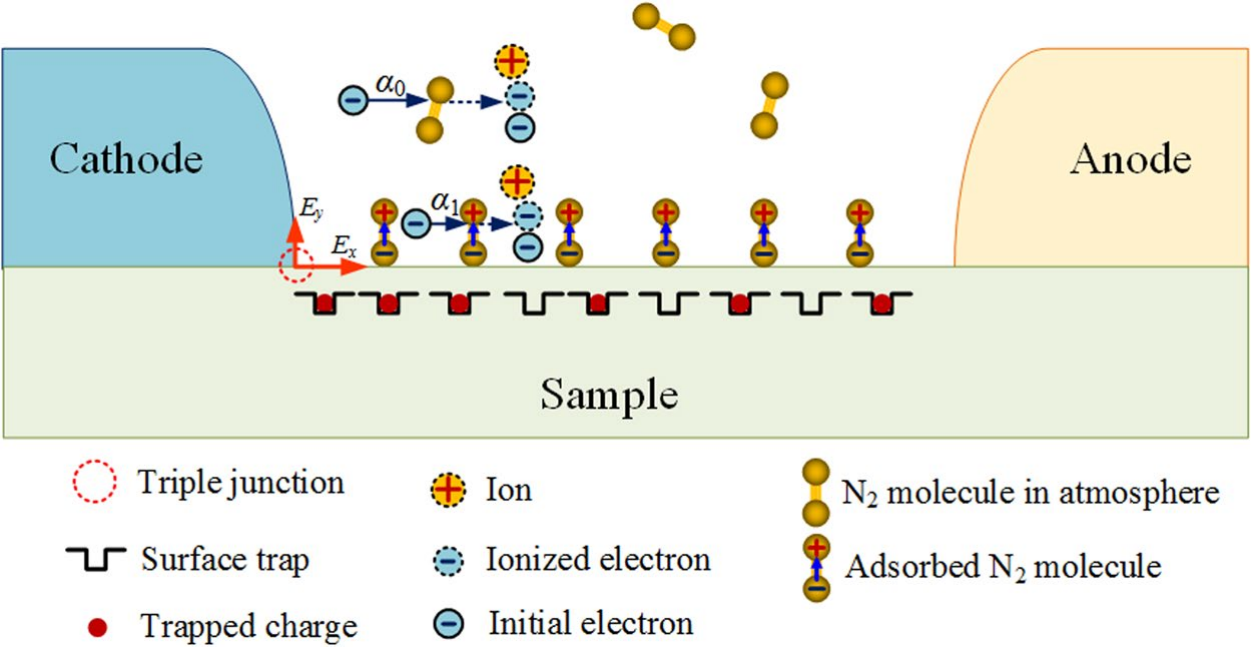
Schematic of surface flashover model. *α*_1_ and *α*_0_ represent impact ionization in adsorbed gas near solid surface and gas space away from the surface, respectively. *E*_x_ and *E*_y_ are tangential and longitudinal electric fields on solid surface, respectively. Solid line with arrow represents the impact of initial electrons with gas molecules, while broken line with arrow represents the production of ionized electrons and ions after impact ionization.

Due to the gas-solid interaction, the surface charging process is altered correspondingly. The surface potential is directly related to the induced electron density of the adsorbed gas molecules, which in turn modifies the electric field distribution across the surface. The captured electrons accumulate at the triple junction point near the cathode, which alleviates the local electric field strength, leading to a lower primary electron emission. The relationship between the adsorbed gas molecules and surface charging is also clearly seen in the gas pressure dependence as shown in Fig. 8. With the increase in pressure, the surface charge density increases, resulting in a higher *V*_f_. Furthermore, the surface traps serve as active centers for attracting electrons. In Fig. 9, the flashover process is schematically shown, emphasizing the important discharging channels induced by the adsorbed gas molecules.

## Conclusion

We investigated the characteristic of gas adsorption on solid surface, and its effect on electrostatic potential distribution of solid surface atoms and gas molecules, by considering the interaction between adsorbed gas and solid.

Results suggest that the quantity of N_2_ adsorbed on epoxy resin is larger than that on Al_2_O_3_ ceramic, and former surface flashover voltage is correspondingly larger than latter one. Also, we found that the relative gas adsorption increases with the decrease in gas pressure, and the ratio of flashover voltage to gas breakdown voltage increases with an increase in the relative gas adsorption. In addition, electron density of the adsorbed gas, surface trap characteristics of the solid and their interactions are influenced by electrostatic potential, so that adsorbed gas and surface traps have the ability to capture electrons, leading to the change in electric field distribution. Furthermore, electron transport in adsorbed gas and solid surface will be affected by electric field, causing the variation in impact ionization and propagation of electron avalanche, and eventually resulting in the occurrence of surface flashover. Based on unique experimental finding and analysis, we proposed a novel surface flashover model by considering the synergic effect of adsorbed gas and charging on surface flashover. The gas adsorption capability on solid surface can be improved by optimizing solid species, surface characteristics, and even the molecular structure of solid, and then improve the surface flashover performance of gas-solid system.

## Methods

### Materials

There are two kinds of samples selected in this paper, which are Al_2_O_3_ ceramic and epoxy resin. The Al_2_O_3_ ceramic sample with a diameter of 35 mm and a thickness of about 4.71 mm is supported by China Ceramic Research Institute. The epoxy resin samples were prepared in the laboratory. The epoxy resin, acid anhydride and accelerator were mixed at a ratio of 100:80:1. The mixture was stirred by a THINKY mixer for 20 min, and defoamed for 15 min. Then, the mixture was poured into a preheated mold, and solidified in a high temperature oven. It was cured at 80 °C for 4 hours, then at 120 °C for 8 hours, and demolded after curing. The epoxy resin sample has a diameter of 50 mm and a thickness of 1 mm. The Al_2_O_3_ ceramic and the epoxy resin sample were cut at a speed of 160 r/min with a SYJ-160 diamond cutter. The sample powder cut by the blade during the cutting process was collected and used as a sample for the isothermal adsorption measurement. Sheet-like Al_2_O_3_ ceramic and epoxy resin samples and their powder samples were dried in an oven at 60 °C for 24 h before all the measurements.

### Adsorption measurements

The isothermal adsorption curve was tested using the fully automated physical adsorber ASAP 2020 Plus HD88. A certain mass of the powder sample was placed in the sample tube, and vacuum degassing was performed at 80 °C for 20 h. After the degassing was completed, isothermal adsorption test was carried out at a room temperature (25 °C). Nitrogen was used as an adsorbate to obtain an isothermal adsorption curve of the sample.

There is an adsorption field on the surface of the adsorbent. When adsorbate molecules are adsorbed, adsorption work (adsorption potential) is required^21^. The adsorption force between the adsorbate molecules and the surface of the adsorbent decreases as the distance between the adsorbate and the adsorbent surface increases.

### Surface flashover measurements

The dc breakdown measurement in dry air, N_2_ and SF_6_ was carried out using a sphere-shpere electrode. The diameter of the electrode is 50 mm, the electrode spacing is 4.71 mm, and the gas pressure is varied from 0.1 Pa to 300 kPa for air, and from 1 Pa to 100 kPa for N_2_ and SF_6_.

The dc surface flashover measurement of ceramic Al_2_O_3_ and epoxy resin samples were conducted in different atmospheres using a fingered type electrode. The front end diameter of the electrode is 20 mm and the electrode spacing is 4.71 mm. The sample diameter is 35 mm. The chamber pressure varies from 1 kPa to100 kPa.

The surfaces of samples and electrodes are polished before the experiment in order to eliminate the influence of surface roughness on gas breakdown and surface flashover. The roughness range of Al_2_O_3_ ceramic, epoxy resin, sphere electrode and the lower surface of fingered type electrode is 1.4-1.6 μm, 1.2-1.5 μm, 4.3-4.4 μm and 3.9-4.0 μm, respectively.

### Simulation of gas adsorption on solid dielectrics

Molecular models of epoxy resin were constructed using the Amorphous Cell module of Material Studio®, with the weight ratios of bisphenol A-type epoxy resin (C_21_H_24_O_4_) and phthalic anhydride (C_8_H_4_O_3_) is 5:4. MD simulations and the energy minimizations were carried out using the Forcite Plus package. The COMPASSII^23^ force field was used to describe the interactions between atoms. The van der Waals (vdW) and electrostatic forces were handled by the Lennard-Jones 9-6 potential and Columbic term, respectively. A NPT process was performed with 400 ps under 0.0001 GPa and 298 K after annealing, ended with an equilibrium configuration with size of 50.12Å × 50.12Å × 29.35Å and density of 1.185 g/cm^3^, which is close to the experimental value of about 1.2 g/cm^3^. The grand canonical Monte Carlo (GCMC) method was employed to calculate the isotherm adsorption curve at 298 K and the probability distribution function of adsorption energy of SF_6_ on the surface of epoxy resin by the Sorption software. From gas pressure of 2.0 kPa to 200.0 kPa, 8 × 10^6^ MC steps were performed, with translation, rotation, insertion, and deletion processes to obtain equilibrium at each pressure.

### Calculated electronic structure of gas-solid system

The adsorption of N_2_ molecule on the α-Al_2_O_3_ (0001) surface were investigated by the density functional theory (DFT) under the Cambridge Serial Total Energy Package (CASTEP)^24^. Our calculations employed a supercell model represented with 2 × 2 of periodic boundary conditions, and reserved a free space about 25Å for a N_2_ molecule above the α-Al_2_O_3_ (0001) surface. The exchange and correlation of electrons were described by the Perdew-Burke-Ernzerhof (PBE) functional^25^ under the generalized gradient approximation (GGA) method^26^. To correct DFT for missing non-bonded interactions, Grimme correction method was applied to consider dispersions contributions (DFT + D) in this adsorption system^27,28^. The cutoff energy of plane waves was set to 570.0 eV. The Monkhorst-Pack scheme^29^ K-points grid sampling was used with mesh parameters of 3 × 3 × 1 for the irreducible Brillouin zone. For geometry optimization and energy calculation, the convergence criterion of energy change, maximum force, and maximum displacement were set at 1 × 10^−6^ eV, 0.03 eV/Å, and 1 × 10^−3^Å, respectively. In the case of N_2_/α-Al_2_O_3_(0001) system, the charge density difference can be expressed as

1 $${\rm{\Delta }}\rho ={\rho }_{{{\rm{N}}}_{2}/\alpha -{{\rm{Al}}}_{2}{{\rm{O}}}_{3}(0001)}-{\rho }_{{{\rm{N}}}_{2}}-{\rho }_{\alpha -{{\rm{Al}}}_{2}{{\rm{O}}}_{3}(0001)}$$

where *ρ*_N2/α-Al2O3(0001)_ is the electron density of the total N_2_/α-Al_2_O_3_(0001) system, and *ρ*_N2_ and *ρ*_α-Al2O3(0001)_ are the unperturbed electron densities of the N_2_ and α-Al_2_O_3_(0001), respectively.

### Calculation process of surface flashover voltage

The calculation program of Matlab^®^ is used to calculate surface flashover voltage and surface charge distribution of the material when the flashover occurs.

## Supplementary information

<b>Supplementary Information</b>

## Data Availability

All data are available.
