# Supplementary material for: Synergic effect of adsorbed gas and charging on surface flashover
Source: Sci Rep. 2019 Apr 2;9:5464. doi: 10.1038/s41598-019-41961-0 (PMC6445122; doi:10.1038/s41598-019-41961-0)
Supplement: Supplementary file 1 — Supplementary Information [file 41598_2019_41961_MOESM1_ESM.docx]

**Supplementary Information**

**Synergic effect of adsorbed gas and charging on surface flashover**

Shengtao Li, Yin Huang, Daomin Min, Guanghao Qu, Huan Niu, Zhen Li,

Weiwang Wang, Jianying Li, Wenfeng Liu

State Key Laboratory of Electrical Insulation and Power Equipment, Xi'an Jiaotong University, Xi'an 710049, China.

Correspondence and requests for materials should be addressed to S. L. (email: sli@mail.xjtu.edu.cn)

**Simulation of gas adsorption on solid dielectrics.** The temperature and pressure of the simulation system were controlled by the Nosé-Hoover thermostat ^1,2^ and Berendsen method ^3^, respectively. We used the crosslinking-relaxation method ^4^ to obtain an initial mixing structure of epoxy resin, with the degree of crosslinking is 88%. Then a high temperature annealing with 20 cycles under NVT ensemble was followed, totally 1.2 ns. The temperature was first raised up to 600 K and dropped to 298 K. Free space about 300 Å was added in the direction perpendicular to the surface of the epoxy resin for gas molecules, which is shown in Fig. S1.


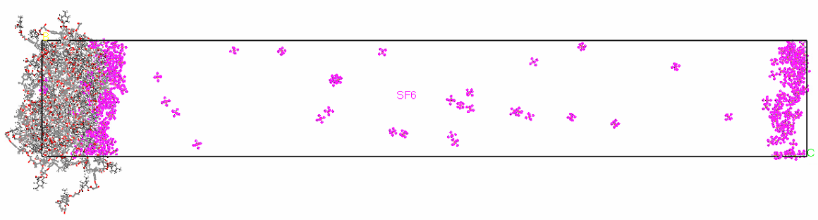


**Figure S1.** The adsorption system for epoxy resin and SF_6_ gas.

Fig. S2 shows the probability distribution curve of SF_6_ adsorbed on neat epoxy resin. As the gas pressure increases, the percentage of adsorbed gas on the surface of the epoxy resin decreases, and the un-adsorbed gas rises. The probability remains almost unchanged after the pressure is larger than 150 kPa.


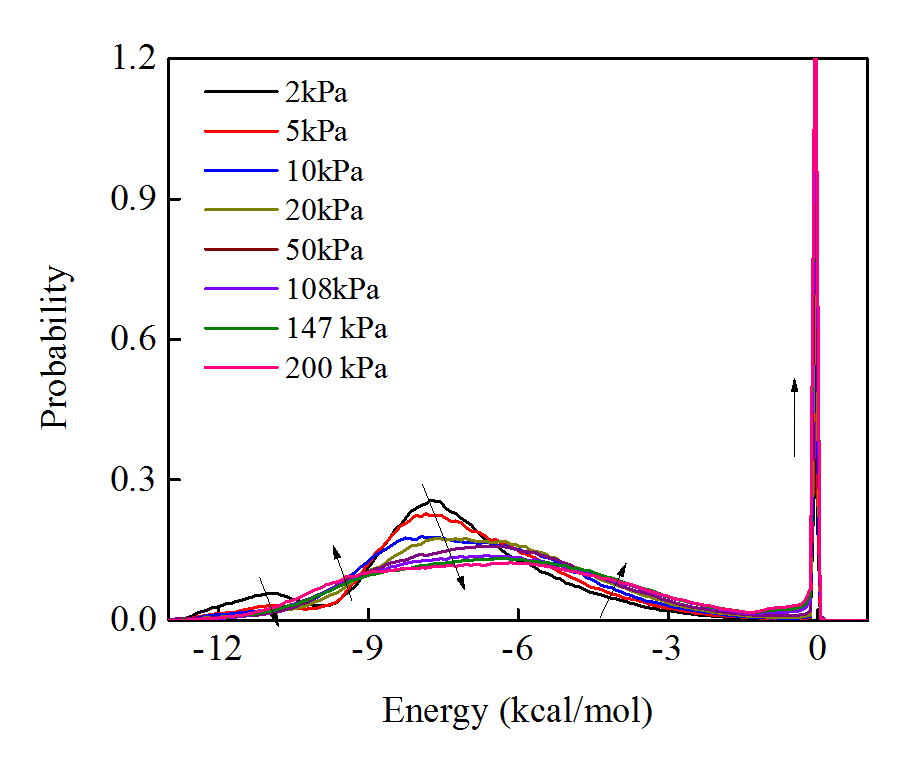


**Figure S2.** The probability distribution function of the adsorption energy of SF_6_ on neat epoxy resin. The direction of the arrow indicates an increase in pressure.

Fig. S3 illustrates a plot of the concentration of SF_6_ gas at room temperature (25 ^o^C) as a function of distance from the neat epoxy resin surface. It can be seen that as the distance increases, the gas concentration shows a tendency to decay. As the gas pressure increases, the relative adsorption amount of SF_6_ on neat epoxy resin in the range of 1 nm decreases.


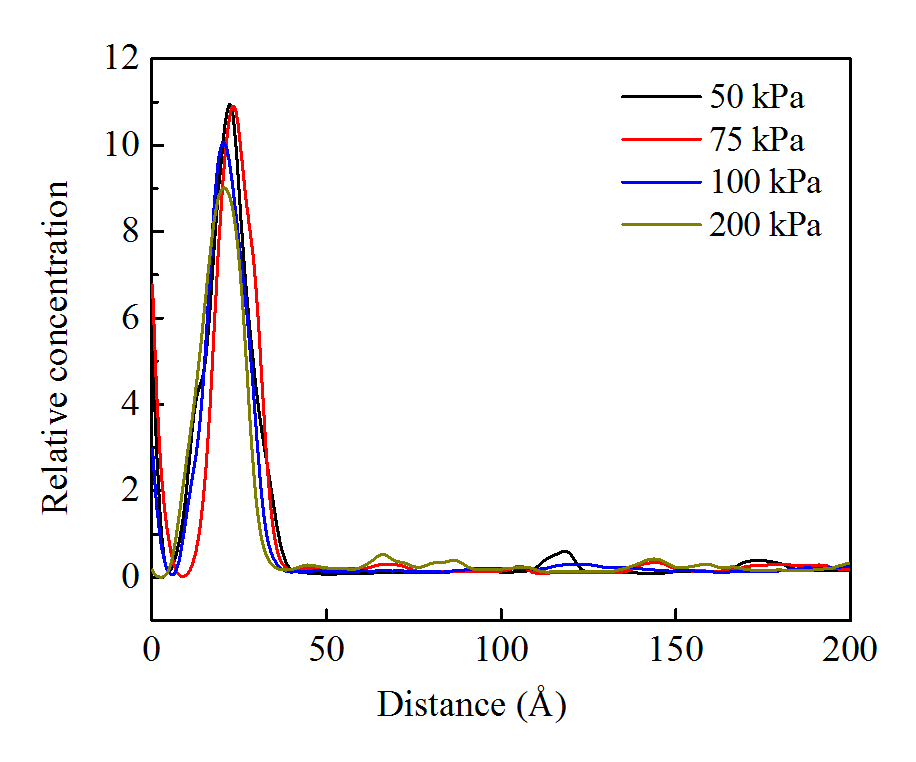


**Figure S3.** The relative adsorption amount for SF_6_ on epoxy resin as a function of distance at room temperature.

**Calculation process of surface flashover voltage.** The main calculation process includes 7 steps. 1) Set initial parameters, such as sample thickness, sample dielectric constant, initial surface charge density, applied voltage. 2) Mesh discretization. The tangential direction of the sample is divided into 50 equal parts. The internal longitudinal direction of the sample is divided into 50 equal parts. The longitudinal direction of the sample is divided into 50 equal parts. The time is divided into 100 equal parts. 3) The grid point value is initialized. The charge density and potential of the grid center are set to zero matrix. The electric field of the grid center along the tangential and longitudinal directions is set to zero matrix. The current density of the grid center along the tangential and longitudinal directions is set to zero matrix. 4) Considering the charge transport process of the surface layer of the material. The current density and surface charge density of the surface layer are calculated by using the electron injection equation at triple injection point, the electron conduction equation and the electron trapping and detrapping dynamic equation. 5) Recalculate the potential, electric field and surface charge density. According to the applied voltage, the initial calculated current and the surface charge density, Poisson’s equation is used to solve the grid and the boundary inside and above the sample to obtain the potential distribution. The potential matrix is ​​solved by the differential to obtain the electric field matrix of the grid. The conduction equation and the current continuity equation are used to calculate the charge density. 6) Calculate the surface flashover voltage.

**References**

1. Nose, S. A unified formulation of the constant temperature molecular dynamics methods. *J. Chem. Phys.* **81,** 511-519 (1984).
2. Hoover, W. G. Canonical dynamics: equilibrium phase-space distributions. *Phys. Rev. A* **31,** 1695-1697 (1985).
3. Berendsen, H. J. C., Postma, J. P. M., van Gunsteren, W. F., DiNola, A. & Haak, J. R. Molecular dynamics with coupling to an external bath. *J. Chem. Phys.* **81,** 3684 (1984).
4. Yang, S. & Qu, J. Computing thermomechanical properties of crosslinked epoxy by molecular dynamic simulations. *Polymer* **53,** 4806-4817 (2012).
